# The quality assessment of intraabdominal infection guidelines/consensuses in 2 decades - which are better and any changes?

**DOI:** 10.1097/MD.0000000000023643

**Published:** 2020-12-11

**Authors:** Yu Wang, Jun Guo, Tingting Xiong, Fangfang Wang, Guoxian Kou, Hong Ning

**Affiliations:** a Department of Pharmacy; b Department of Pediatrics, MianYang Center Hospital, MianYang; c Department of Pharmacy, sichuan provincial hospital for women and children, Chengdu; d Department of Pharmacy, Yibin hospital for women and children, Yibin; e Department of Infectious Diseases; f Department of Pharmacy, MianYang Center Hospital; g Northwestern SiChuan Regional Medical Center, MianYang, China.

**Keywords:** AGREE II, guidelines/consensuses, Intraabdominal infection, quality assessment

## Abstract

**Background and aim:**

Intraabdominal infection (IAI) is a common and important disease worldwide. An increasing number of related guidelines/consensuses have been published in recent years, the quality evaluation for these guidelines/consensuses is necessary to identify lower-quality documents and explore the quality distribution in different time range and areas in this field.

**Methods:**

The Appraisal of Guidelines for Research & Evaluation Instrument tool was adopted to assess the quality of IAI guidelines/consensuses by 3 researchers independently. Intraclass correlation coefficients (ICCs) among the researchers were retrieved to reflect reliability. The quality differences of these guidelines/consensuses issued before and after May 2009, both international and non-international, were compared by a Mann–Whitney *U* test.

**Results:**

Fourteen IAI guidelines/consensuses published in English were obtained following a literature search. The ICCs among the researchers were all above 0.75, indicating satisfactory reliability. This outcome showed that the overall quality of these guidelines/consensuses was mediocre and considered acceptable in all items. A few guidelines/consensuses were better in their scientific and methodological characteristics than the others. Moreover, there were no significant differences in the scores between the guidelines/consensuses issued before and after May 2009 or between international vs regional guidelines/consensuses.

**Conclusions:**

Overall, the quality of the IAI guidelines/consensuses was generally acceptable and applicable, with a few deficiencies. Therefore, continuous improvement is essential. The guideline assessment tools should be applied in guideline/consensus development both widely and strictly to improve the methodological quality.

## 1 Introduction

Intraabdominal infection (IAI) is a common problem worldwide. It can be caused not only by gastrointestinal fistula, abdominal multiple trauma, biliary tract diseases, and appendicular diseases but also by abdominal surgery and operation. It is considered the third most commonly identified cause of sepsis and the second most common cause of death in the intensive care unit.^[1]^ Therefore, to improve the diagnosis and treatment of IAIs, some professional organizations have developed guidelines/consensuses as part of their responsibilities. Overall, the guidelines/consensuses have been developed with the aim of bridging the gap between research and clinical practice; however, issues still remain.^[2]^ The guidelines/consensuses on IAIs may be of different quality as they were compiled by various institutions and professionals from varied nations and areas with different methodologies and professional backgrounds. Therefore, it is necessary to assess the quality of these guidelines/consensuses, which is important for selecting and applying a better guidelines/consensuses in clinical settings. This process may also affect the quality of the future guidelines/consensuses. Another matter is that with the trend of evidence-based guidelines development, whether the quality of guidelines/consensus in this field has been improved, and whether the quality of these documents in different regions are various?

The Appraisal of Guidelines for Research & Evaluation Instrument II (AGREE II) is an important guideline evaluation tool published by the AGREE cooperative group. It uses a detailed framework to assess guideline quality but also provides a methodological strategy for guideline development and content.^[3]^ Moreover, it can also be used as a tool for evaluating the quality of the consensuses and the position statement.^[4]^ This system is considered to be accurate and rigorous. Given the advantage of the AGREE II tool, it has been adopted by the World Health Organization for the assessment of guidelines and is widely used in other fields as well.^[5]^

To date, few researchers have focused on this subject. This is obviously not beneficial to enhancing quality improvement and clinical application. Therefore, our research focuses on the last 20 years of this field.

## 2 Methods

### 2.1 Eligibility criteria and search strategy

**Figure 1:**
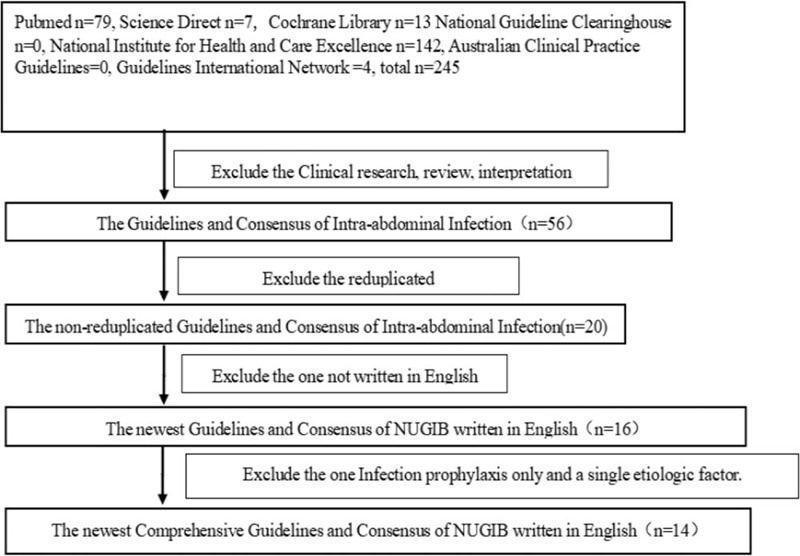
The process of the literature search. “n” indicate the quantity of literatures.

The guidelines/consensuses were searched from 01/2000 to 02/2020 in PubMed, the Cochrane Library, Science Direct, National Guideline Clearing House (www.guideline.gov), National Institute for Health and Care Excellence (www.nice.org.uk.com), Australian Clinical Practice Guidelines (http://www.clinicalGuidelines.gov.aur.r.), and Guidelines International Network (http://www.g-i-n.net). The search terms were “intraabdominal, intra-abdominal, infections, infection and guideline, guidelines, guidance, consensus, statement, statements, positions or position”. The exclusion criteria were single pathogens such as IAIs caused by organ transplant only, guidelines/consensuses with infection prevention, redundant publications of the same edition in different databases and documents published not in English. The process of the literature search is shown in Figure 1.

### 2.2 Data extraction

The data on the number of references and editors, publication organizations, country or area, time of publication and the methodology of the clinical evidence evaluation for the guidelines/consensuses were extracted in Table 1.

**Table 1:** Characteristics of the retrieved clinical guidelines/consensuses.

Names of guidelines and consensuses	Type	Page	Institutions	Authors number	Whether update version or not	Number of references	Year of publication	Evidence evaluation tool	Countries and Regions
WSES consensus conference: Guidelines for firstline management of intra-abdominal infections	Guidelines	29	The World Society of Emergency Surgery	16	NO	278	2011	Grade	World
2013 WSES guidelines for management of intra-abdominal infections	Guidelines	29	The World Society of Emergency Surgery	55	YES	274	2013	Grade	World
Management of intra-abdominal infections	consensus	31	The World Society of Emergency Surgery	39	NO	284	2016	Grade	World
The management of intra-abdominal infections from a global perspective: 2017 WSES guidelines for management of intraabdominal infections	guidelines	34	The World Society of Emergency Surgery	71	YES	304	2017	Grade	World
The Surgical Infection Society Guidelines on Antimicrobial Therapy for Intra-Abdominal Infections	Guidelines	61	Therapeutic Agents Committee of the Surgical In fection Society	7	YES	130	2002	The Canadian Task Force on the Periodic Health Evaluation	America
The Surgical Infection Society Revised Guidelines on the Management of Intra-Abdominal Infection	Guidelines	76	The Surgical Infection Society and the Infectious Diseases Society of America	12	YES	690	2017	Grade	America
Guidelines for the Selection of Anti-infective Agents for Complicated Intra-abdominal Infections	Guidelines	9	The Infectious Diseases Society of America	12	NO	71	2003	IDSA–United States Public Health Service grading system	America
Diagnosis and Management of Complicated Intra-abdominal Infection in Adults and Children: Guidelines	Guidelines	32	The Surgical Infection Society and the Infectious Diseases Society of America	16	YES	189	2009	Canadian Task Force on the Periodic Health Examination	North America
Canadian practice guidelines for surgical intra-abdominal infections	Guidelines	28	The Canadian Surgical Society (CSS) and the Association of Medical Microbiology and Infectious Disease	9	YES	258	2010	Grade	Canada
Guidelines for management of intra-abdominal infections	Guidelines	14	**Société Françaised’Anesthésie et de Réanimation**	18	YES	96	2015	Grade	France
Jávea consensus guidelines for the treatment of Candida peritonitis and other intra-abdominal fungal infections in non-neutropenic critically ill adult patients	Guidelines	13	Servicio de Microbiología, Hospital Universitario y Politécnico La Fe, Valencia, Spain	6	NO	66	2017	NA	Spain
Consensus statement on antimicrobial therapy of intra-abdominal infections in Asia	Consensus	5	Asian Surgical Speakers’ Forum	9	NO	7	2007	NA	Asia
Guidelines for antimicrobial therapy of intra-abdominal infections in adults	Guidelines	3	Infectious Diseases Society of Taiwan; Taiwan Surgical Society of Gastroenterology	38	NO	NA	2008	NA	China taiwan
Clinical Practice Guidelines in Complicated Intra-Abdominal Infection 2018: An Indonesian Perspective	Guidelines	8	Department of Surgery, from Different hospital in Indonesia	9	NO	36	2018	Agree II	Indonesia

^†^NA = not available.

### 2.3 Quality assessment of guidelines/consensuses

Three assessors participated in this research. At first, all the researchers were trained to use the AGREE II tool skilfully to ensure the accuracy of the assessment. Then, they independently responded to 23 questions of 6 domains by a scale ranging from 1 for “strongly disagree” to 7 for “strongly agree”. The total score of each domain was calculated as follows: (the actual score - the lowest possible score)/(the highest possible score - the lowest possible score) ×100%. The 3 scores were averaged. A positive correlation was found between score and guideline quality.^[6]^

There was no specific or distinct threshold of scores in the AGREE II tool when it was updated in 2017. But it suggests that “thresholds can be created based on scores for the prioritized domain through consensus”.^[6]^ A new threshold has been adopted by some institutions or experts^[7–8]^: Grade C (not recommended): ≥3 domains with a score <30%; Grade B (recommended after revision): ≥3 domains of score ≥30%, but at least 1 domain of score <60%; and Grade A (recommended): 6 domains with a score ≥60%. The thresholds could be adapted in the study after a discussion by all researchers.

### 2.4 Statistical analysis

The intraclass correlation coefficient (ICC) is used to assess the consistency or conformity between 2 or more quantitative measurements.^[9]^ This method has been widely used to evaluate the differences in results between various researchers. In observational studies, when the ICC is close to 1, repeated measurements from a particular individual are expected to remain consistent.^[10]^ Specifically, the consistency is considered sufficient when the ICC ≥0.75, indicating that the differences in the results between the researchers are acceptable. Furthermore, to identify whether the intraclass correlation coefficient is significant, the ICC must been tested by an analysis of variance; the standards above are available only when *P* < .05.^[11]^ A nonparametric test (Mann–Whitney *U* test) was used for the analysis of the differences in the scores between guidelines/consensuses issued before and after May 2009 for international and regional documents. The differences were significant at *P* < .05. All analyses above were performed in Statistical Product and Service Solutions (SPSS) 23.0. The functions of SPSS 23.0 include data management, statistical analysis, chart analysis, and so forth. The statistical analysis process include but not limited to descriptive statistics, hypothesis test, and correlation analysis.

## 3 Results

### 3.1 Guideline characteristics

Among the 14 documents included were 12 guidelines and 2 consensuses (Table 1).^[12–26]^ The promulgation time ranged from 2002 to 2018. The content ranged from 3–76 pages, 7–690 references, and 7–55 authors participating in the editing. Eight document was issued by the Infectious Diseases Society or Surgical Society separately, 2 was by the Anaesthesia and Reanimation Association or medical college. Notably, 4 documents were issued by the Surgical Society in collaboration with the Infectious Diseases Society. As a whole, most documents adopted the “GRADE” evidence evaluation system except the earlier and non-Western ones. Remarkably, Indonesia 2018 applied the “AGREE” system as a tool to evaluate the other guidelines to build a guidance document for their nation based on the advantages of other documents, but offered No details.

### 3.2 Consistency assessment

The ICCs of all the guidelines/consensuses among the researchers were ≥0.75 (*P* < .05), illustrating that the consistencies of the different research results were satisfactory and acceptable (Table 2).

**Table 2:** The Consistency assessment of the guidelines /consensuses evaluation by different researchers.

	ICC	1CCMIN	ICCMAX	F	*P*
WSES2011^[12]^	0.97	0.94	0.98	36.58	.000^∗∗∗^
WSES2013^[13]^	0.97	0.94	0.99	39.19	.000^∗∗∗^
WSES2016^[14]^	0.98	0.95	0.99	42.10	.000^∗∗∗^
WSES2017^[15]^	0.97	0.94	0.99	41.00	.000^∗∗∗^
SIS 2002^[16,17]^	0.98	0.96	0.99	48.50	.000^∗∗∗^
SIS 2017^[18]^	0.94	0.88	0.97	17.62	.000^∗∗∗^
IDSA2003^[19]^	0.97	0.94	0.99	43.66	.000^∗∗∗^
IDSA2009^[20]^	0.98	0.96	0.99	43.90	.000^∗∗∗^
Canada 2010^[21]^	0.97	0.94	0.99	34.25	.000^∗∗∗^
French 2015^[22]^	0.98	0.96	0.99	43.55	.000^∗∗∗^
Spain 2017^[23]^	0.96	0.92	0.98	24.14	.000^∗∗∗^
Asia 2007^[24]^	0.97	0.94	0.99	34.73	.000^∗∗∗^
China-taiwan2008^[25]^	0.96	0.91	0.98	22.91	.000^∗∗∗^
Indonisia2018^[26]^	0.91	0.81	0.96	10.55	.000^∗∗∗^

^†,∗^*P* < .05,^∗∗^*P* < .01,^∗∗∗^*P* < .001.

### 3.3 AGREE II scores

The details and total score of each domain were worked out by the AGREE II tool. Two guidelines (IDSA 2009, SIS 2017) were strongly recommended for use with a higher overall quality as a result of 3 item scores being above 60%. The scores of Asia 2007 and China–Taiwan 2008 had no item above 60%, and the majority of items were <30%, demonstrating a lower quality (Table 3).

**Table 3:** The score and recommendation level of the guidelines /consensuses by AGREE II.

Names of guidelines / consensus	Scope and purpose	Stakeholder involvement	Rigor of development	Clarity of presentation	Applicability	Editorial Independence	NO of score ≥ 60%	NO of score < 30%	Grades of recommendations
WSES2011	40.74%	20.37%	26.39%	72.22%	16.67%	33.33%	1	3	C
WSES2013	42.59%	14.81%	27.78%	72.22%	16.67%	30.56%	1	3	C
WSES2016	51.85%	16.67%	31.94%	75.93%	16.67%	63.89%	2	2	B
WSES2017	53.70%	16.67%	31.94%	75.93%	27.78%	69.44%	2	2	B
SIS 2002	74.07%	12.96%	55.56%	75.93%	26.39%	0.00%	2	3	C
SIS 2017	81.48%	31.48%	57.64%	81.48%	41.67%	72.22%	3	0	B
IDSA2003	85.19%	42.59%	36.11%	59.26%	16.67%	36.11%	1	1	B
IDSA2009	79.63%	16.67%	52.78%	75.93%	13.89%	72.22%	3	2	B
Canada 2010	59.26%	22.22%	46.53%	75.93%	13.89%	63.89%	2	2	B
French 2015	70.37%	37.04%	47.92%	81.48%	12.50%	0.00%	2	2	B
Spain 2017	51.85%	16.67%	15.28%	61.11%	6.94%	47.22%	1	3	C
Asia 2007	12.96%	14.81%	10.42%	40.74%	2.78%	33.33%	0	4	C
China-taiwan 2008	27.78%	20.37%	5.56%	55.56%	15.28%	0.00%	0	5	C
Indonisia 2018	64.81%	33.33%	20.83%	70.37%	9.72%	27.78%	2	3	C

### 3.4 The quality of guidelines/consensuses distribution by time and region

There were no significant differences in the scores between the guidelines/consensuses issued before and after May 2009 or between the international and regional guidelines/consensuses (Table 4).

**Table 4:** The quality of guidelines / consensuses distribution in various time and regional.

Names of guidelines / consensus	Scope and purpose	Stakeholder involvement	Rigor of development	Clarity of presentation	Applicability	Editorial independence
Worldwide Non-Worldwide	*P* = .142 Z = 30.5	*P* = .304 Z = 28.0	*P* = .635 Z = 24.0	*P* = .733 Z = 17.0	*P* = .106 Z = 8.5	*P* = .635 Z = 16.0
Pre-2009 may Post-2009 may	*P* = .839 Z = 22.0	*P* = .635 Z = 24.0	*P* = .539 Z = 25.0	*P* = .054 Z = 34.0	*P* = 1.000 Z = 20.5	*P* = .106 Z = 31.5

^†,∗^*P* < .05,^∗∗^*P* < .01,^∗∗∗^*P* < .001.

## 4 Discussion

The 14 published guidelines/consensuses were mainly from Europe, North America, and Asia. Guidelines/consensuses from Africa and South America were rare. The overall quality of these guidelines/consensuses was moderate in all items but better in the domains of “scope and purpose and clarity of presentation” and weaker in the domains of “stakeholder involvement and applicability”. Notably, a few guidelines/consensuses were well developed with better scientific and methodological quality, but some of the others had obvious defects.

### 4.1 Scope and purpose

The AGREE II tool is required to make the theoretical and practical significance explicit before editing the guidelines/consensuses. To avoid the inadequate use of the guidelines/consensuses, showing the specific target population is required. In this domain, the majority of guidelines/consensuses performed well, especially in the guidelines issued by the Infectious Diseases Society and the Surgical Infection Society of America. These associations devote much effort to explaining the scope and purpose clearly in the guidelines. Even in the updated version, the new focal points were also emphatically mentioned to prompt the readers to pay attention to the changes in purpose at the same time. The scope of these documents were clearly defined as complex abdominal infection. In particular, they could not be applied to the treatment of primary peritonitis, catheter-related peritonitis, et al. Misuse of the guidelines by doctors could be avoided to some extent. On the other hand, there were more defects mainly concerning the lack of some essential elements in the guidelines/consensuses of Asia 2007 and China–Taiwan 2008. More attention should be paid to this aspect.

### 4.2 Stakeholder involvement

Regarding this item, the overall quality was not satisfactory compared with the other items. All guidelines can be improved in this regard. This item was mainly associated with the points of the target population about the documents, that is, whether the target users and the professional integrity of the expert group members were demonstrated. IDSA 2003 listed the explicit target audience. However, the others rarely mentioned it. Notably, few documents paid attention to the opinions of the target population. Thus, whether the diagnosis and treatment are conducive to the establishment of patient compliance is still uncertain. The same regrettable situation is most guidelines/consensuses lacked the participating of statistician and methodologist. This lack may passively affect the framework and evidence quality evaluation standards of the guidelines/consensuses. Moreover, intraabdominal infection is a disease that requires physicians and surgeons to work together, as surgical debridement and medical treatment are often both necessary. However, some documents were compiled by experts from only 1 field mainly, making some recommendations tendentious and not objective.

### 4.3 Rigor of development

Trustworthy guidelines can help guide clinicians and patients in making decisions on appropriate care in specific circumstances and potentially improve the outcomes of patients.^[27]^ The reliability of the recommendations based on evidence could be the most important factor for whether the guidelines/consensuses are accepted and trusted widely. Two factors appear to be essential in the field. First, the search and evaluation process of the evidence should be objective and repeatable. Thus, the guidelines/consensuses must provide the inclusion or exclusion criteria, and search or assessment process descriptions. Second, the recommendations should be explicitly supported by the current clinical evidence. In this regard, the performance of these documents were generally acceptable but still seen as unsatisfactory. Some guidelines/consensuses (WSES 2011, 2013, 2016, 2017, Asia 2007, China–Taiwan 2008, Spain 2017) did not refer to information on the evidence collection and estimate. In the process of formulating the recommendations, most of the guidelines/consensuses described methods for the formation of recommendations. Similarly, they also announced the health benefits, side effects, and risks of the recommendations. However, some documents did not describe these elements clearly. In addition, in terms of the links between the evidence and recommendations, the majority of these documents could comply with the rule that the recommendations be supported by adequate evidence if found, but a few appeared to ignored it (China–Taiwan 2008, Indonesia 2018).

In addition, the external expert review was not noted clearly in most of the guidelines/consensuses except IDSA 2009, SIS 2017, France 2015, and Canada 2010, implying the shortage of peer supervision. Furthermore, most of them lacked innovation plans, indicating that the update of these guidelines/consensuses seem to lack sustained motivation.

### 4.4 Clarity of presentation

Regarding this item, the recommendations were all specific and unambiguous in terms of descriptions that will not mislead the readers. Most of the documents also provided different recommendations according to the severity of the infection and location of disease (community or hospital-acquired infection). And they also distinguished between the recommendations for surgical treatment and non-surgical treatment.

### 4.5 Applicability

The scores of all guidelines/consensuses in this item were lower overall. The main problem was that most of the editors neglected to investigate the effect of potential resource implications that could improve the implementation of the recommendations. Considering the imbalance of economic and social development in different countries and regions, suggestions on potential resource input by the guidelines/consensuses should be made. Moreover, some have still ignored to describe the monitoring and/or auditing criteria. This may lead to undesirable consequences. For example, sepsis is a common complications of IAIs, but its definition has been changed in the past several years.^[28]^ Some guidelines/consensuses have listed the definition adopted by the documents, but some have not. This may cause inconsistencies in the diagnosis and treatment of the IAI sepsis patients.

### 4.6 Editorial independence

Most guidelines/consensuses declared the conflicts of interest among different staff, but some did not mention the financial or other supports by academic or commercial institutions during the process of document development. It is necessary to improve these aspects in future work.

### 4.7 The quality of guidelines/consensuses distribution in various times and geographic scales

The issuing of the AGREE tool in May 2009 did not seem to affect the quality of these documents in all domains, the differences in scores before and after May 2009 were not significant. This outcome indicates that the AGREE tool was not used well when the documents were developed. Another observation is that the quality of international guidelines/consensuses does not seem to be better than the regional ones. In general, international guidelines/consensuses may have higher quality because in theory, the international organizations can mobilize more professional resources. One other study also found that the international guidelines may be unsatisfactory in some domains.^[29]^ Regionality appeared to have little effects on the quality of the guidelines. These phenomena are expected to be refined completely or in part in updated versions.

### 4.8 Limitations of this study

This study examined the quality of documents published in English only; guidelines/consensuses in non-English languages (such as Spanish and Turkish) were not included. This approach may have a disadvantageous impact on the comprehensiveness of the study. Moreover, the AGREE II tool only addresses the formation and quality of the evidence and not its authenticity or accuracy.^[30]^

## 5 Conclusions

In conclusion, the overall quality of the IAI guidelines/consensuses in the past 20 years was generally acceptable and applicable. However, deficiencies remain. Some guidelines/consensuses have lower scores for some items, indicating the necessity of improving these fields. The recommendation of the guidelines/consensuses with higher scores may be recognized as a priority in certain conditions. Moreover, the guideline assessment tools should be applied in guideline/consensus development widely to improve the methodological quality.

## Acknowledgments

It is grateful to Dr Zhou Mengjun (The statistician) for the support of the statistical consultation and the review of the statistical methods in this manuscript.

## Author contributions

Following authors have made substantial contributions to the manuscript as under:

**Fangfang Wang:** The quality of the guidelines/consensuses assessment

**Guoxian Kou:** The consultant of infection disease; The rate of progress supervision

**Hong Ning:** Research design; The consultant of Pharmacy; The rate of progress supervision

**Jun Guo:** The quality of the guidelines/consensuses assessment; Data statistics

**Tingting Xiong:** Research design; Literature search and collection; Manuscript creation

**Yu Wang:** Research design; Literature search and collection; The quality of the guidelines/consensuses assessment; Data statistics; Manuscript creation
